# Notes of M. Bernard’s Lectures on the Blood

**Published:** 1854-12

**Authors:** 


Art. X.—*Notes of M. Bernard's Lectures on the Blood; with an Appendix.* By Walter F. Atlee, M. D. Philadelphia: Lippincott, Grambo & Co. 1854. pp. 224, 12mo.

M. Bernard has a reputation among the working physiologists of the day not exceeded by any other. His experiments and discoveries have been generally published in the journals, and have attracted great attention. The little work before us comprises his lectures on the blood delivered a year ago, in Paris, and taken down by the Editor, Dr. Atlee, a promising young American physician. We thank Dr. Atlee for the opportunity afforded us of seeing the last word of French science on this interesting subject. We shall endeavor at an early day to lay before our readers the prominent points in these lectures. In the mean time, we advise them to avail themselves of the work itself, which will amply repay them for the study they may bestow upon it.

